# An innovative paradigm of composition optimization for nickel-based single-crystal superalloys

**DOI:** 10.1093/nsr/nwaf382

**Published:** 2025-09-11

**Authors:** Lin Liu

**Affiliations:** College of Materials Science and Engineering, Northwestern Polytechnical University, China

Nickel-based single-crystal (SX) superalloys are key materials used for turbine blades. With the increase in turbine inlet temperatures, continuous improvement in the creep rupture life is a major target for developing the next generation of SX superalloys. Currently, the design of SX superalloys primarily depends on the rhenium (Re) and ruthenium (Ru) contents. The addition of Re improves the creep rupture life but promotes topologically close-packed phase precipitations, while Ru improves microstructural stability but increases the cost. Therefore, there appear to be few effective solutions for optimizing the chemistry of ‘next-generation’ SX superalloys. Additionally, designing next-generation SX superalloys through traditional alloying design methods, such as on the basis of trial heat testing, the statistical analysis of experimental data by using regression analysis/neural networks, or in some instances metallurgical rules of thumb derived from experience [1,2], is becoming increasingly challenging.

**Figure 1.**
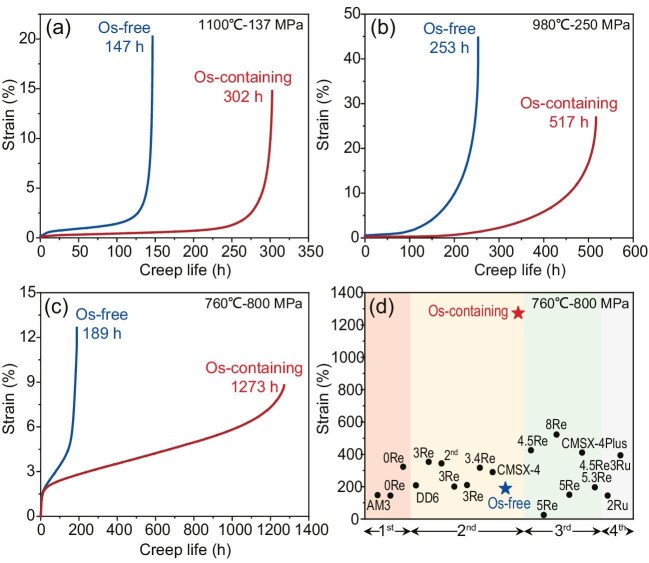
Creep properties of the Os-free and Os-containing superalloys under different conditions. (a–c) Creep curves of the two alloys tested under 1100°C/137 MPa, 980°C/250 MPa and 760°C/800 MPa conditions, respectively. (d) Comparison of the creep life tested under 760°C/800 MPa conditions of the current superalloys in the literature. Reproduced with permission from Ref. [3].

How can the contradiction between the creep performance and structural stability of Re-bearing single-crystal superalloys be solved? Based on their innovative alloy design idea of ‘mixing enthalpy solid solutions’, Professor Han’s team believes that the osmium (Os) element contains P-enthalpy with Ni and N-enthalpy with Al, Ti and Ta, which offer a powerful strengthening effect. A newly developed alloy with 1.0 wt% of the Os element demonstrates remarkable creep performance. Under 1100°C/137 MPa and 980°C/250 MPa test conditions, the creep life of the Os-containing alloy is 302 and 517 h—more than double that of the base alloy without Os additions. Extraordinarily, the creep life of the Os-containing alloy approaches an amazing 1273 h under 760°C/800 MPa conditions—more than six times that of the base alloy, as seen in Fig. 1 [3]. These results set a new benchmark for creep performance for Ni-based SX superalloys. Incorporating this engineered Os-containing alloy into turbine blade materials could greatly enhance aeroengine performance.

The addition of Os significantly improves creep performance through the formation of local chemical ordering (LCO) and segregation at the γ/γ’ interface, which reduces both the γ’ sizes and the γ/γ’ channel width by increasing the stability of the γ’ phase. The mechanism obstructs the dislocation movement from LCO and clusters in the γ phase, and increases microstructural stability during the creep. These optimizations of the alloy chemistry design not only fully comply with the design principles of nickel-based SX superalloys [4], but also break through the preconceptions of traditional composition selection. In recent years, Professor Han’s team has developed new principles for designing metallic materials based on negative enthalpy alloying, providing new paradigms and guidelines for the design of alloys with high strength and ductility [5]. This study sheds new light on the route for materials design through interface engineering and size effects by the mixing enthalpy strategy and N-enthalpy alloying, which promise a novel design concept for high-performance Ni-based SX superalloys.
